# Using Automated Health Plan Data to Assess Infection Risk from Coronary Artery Bypass Surgery

**DOI:** 10.3201/eid0812.020039

**Published:** 2002-12

**Authors:** Richard Platt, Ken Kleinman, Kristin Thompson, Rachel S. Dokholyan, James M. Livingston, Andrew Bergman, John H. Mason, Teresa C. Horan, Robert P. Gaynes, Steven L. Solomon, Kenneth E. Sands

**Affiliations:** * Centers for Disease Control and Prevention Eastern Massachusetts Prevention Epicenter, Boston, Massachusetts, USA; † Harvard Medical School and Harvard Pilgrim Health Care, Boston, Massachusetts, USA; ‡ Brigham and Women’s Hospital, Harvard Medical School, Boston, Massachusetts, USA; § Tufts Health Plan, Boston, Massachusetts, USA; # Blue Cross Blue Shield of Massachusetts, Boston, Massachusetts, USA; ** Centers for Disease Control and Prevention, Atlanta, Georgia, USA; †† Beth Israel Deaconess Medical Center, Boston, Massachusetts, USA

## Abstract

We determined if infection indicators were sufficiently consistent across health plans to allow comparison of hospitals’ risks of infection after coronary artery bypass surgery. Three managed care organizations accounted for 90% of managed care in eastern Massachusetts, from October 1996 through March 1999. We searched automated inpatient and outpatient claims and outpatient pharmacy dispensing files for indicator codes suggestive of postoperative surgical site infection. We reviewed full text medical records of patients with indicator codes to confirm infection status. We compared the hospital-specific proportions of cases with an indicator code, adjusting for health plan, age, sex, and chronic disease score. A total of 536 (27%) of 1,953 patients had infection indicators. Infection was confirmed in 79 (53%) of 149 reviewed records with adequate documentation. The proportion of patients with an indicator of infection varied significantly (p<0.001) between hospitals (19% to 36%) and health plans (22% to 33%). The difference between hospitals persisted after adjustment for health plan and patients’ age and sex. Similar relationships were observed when postoperative antibiotic information was ignored. Automated claims and pharmacy data from different health plans can be used together to allow inexpensive, routine monitoring of indicators of postoperative infection, with the goal of identifying institutions that can be further evaluated to determine if risks for infection can be reduced.

Because postoperative surgical site infections are common complications of medical care, reducing their occurrence is a component of current efforts to improve patient safety. To guide these efforts and to measure their success, hospitals maintain resource-intensive programs to identify these infections (1–4). However, hospital-based programs detect only a minority of these infections. A principal contributor to the poor detection rates of hospital-based systems is the fact that a large majority of infections manifest after the patient leaves the hospital (5–13). Additionally, the substantial resources required to conduct prospective case detection requires some hospitals to monitor specific types of procedures only periodically, which means that hospitals may fail to detect problems that occur while they focus on other procedure types. Finally, variability in application of surveillance criteria has made comparing postoperative infection rates between hospitals difficult (4,14–16).

We have described the use of diagnoses and procedures listed on automated billing data and of antibiotic prescriptions identified through automated pharmacy dispensing data to identify patients who are likely to have experienced postoperative surgical site infection either before or after discharge from the hospital (5,17,18). Overall, insurers’ billing and pharmacy data identified substantially more patients with infection than did hospital-based surveillance in these studies.

These findings suggest that health plans’ and insurers’ routinely collected billing data might be used to supplement hospital-based programs. More importantly, this information might be used to compare different hospitals’ results. However, the comparability of data from different health plans for this purpose has not been assessed. Nor has any attempt been made to combine information from different health plans; information from several health plans is often necessary because the number of procedures for persons in one health plan is usually too small to allow acceptably precise estimates of risk. Health plans differ in type, detail, quality, and completeness of the information they collect and maintain. For example, health plans vary widely in their prescription drug coverage and, therefore, in the amount of information they possess about their members’ antibiotic exposure.

We assessed the usefulness of using data from several large health plans to detect patients who may have had an infection after coronary artery bypass grafting; we wanted to know if this information could be used to compare the experience of different hospitals. Additionally, we assessed the contribution of pharmacy data to these comparisons to determine the potential for using data from persons without pharmacy benefits.

## Methods

### Study Population

We studied patients who had coronary artery bypass graft (CABG) surgery from January 1996 through March 1999 and who received health-care coverage from Harvard Pilgrim Health Care, Tufts Health Plan, or Blue Cross Blue Shield of Massachusetts. Harvard Pilgrim and Tufts Health Plan (including Secure Horizons, Tufts Health Plan for Seniors, a Medicare + Choice managed care plan) are HMOs. Blue Cross included HMO and indemnity plans. Together, these organizations accounted for approximately 90% of managed care in eastern Massachusetts during that time. We focused principally on procedures performed in four hospitals, which were members of the Eastern Massachusetts Prevention Epicenter, and which performed CABG. These institutions performed a majority of the CABG procedures in the region. CABG procedures at Harvard Pilgrim through June 1997 were described in a separate report, and so are omitted here. Patients were excluded who had a second CABG procedure within 30 days of a previous procedure.

Although we wanted to restrict the population to persons with prescription drug coverage, some of the HMOs could not provide coverage status at the time of surgery. Therefore, we excluded from our main analysis any persons for whom the HMO had no claims for any prescription drugs for 180 days before the date of surgery. Among patients for whom pharmacy benefit status was provided, pharmacy dispensing (at least one prescription) 180 days before surgery correlated with having benefits; 97% of those with some dispensing had a drug benefit compared to 19% with no drug benefit.

### Data Sources

#### Automated Data

At the time of the study, Tufts Health Plan maintained two administrative claims systems: one for its commercial plan and another for a Medicare plan. Harvard Pilgrim maintained three legacy systems: one derived from a staff model HMO, one from its network and group division, and one from an IPA system. In total, six claims systems were used. HMO staff searched these six claims systems for hospital claims with ICD9 procedure codes 36.10–36.16, 36.19, or CPT codes 33510–14, 33516–19, 33521–23, 33533–36 in physician billing records. For convenience, we refer to each of these claims systems as a separate plan (e.g., plan 1 through plan 6). These separate divisions had generally similar benefits (i.e., covering ambulatory services and prescription drugs except for a copayment required of the patient). However, one of the plans changed its coverage during the study period, including a capped pharmacy benefit for every 3-month period and a period of no pharmacy coverage.

For all patients with a procedure code of interest, each health plan provided the patient’s age and sex. The health plans also provided inpatient and outpatient claims from the index hospitalization through 30 days after the date of the surgical procedure. For each claim, they provided an encrypted patient identifier, a date of service, a service location (inpatient, skilled nursing facility, emergency room, or outpatient), and all diagnosis and procedure codes. For each prescription, they provided the same patient identifier, the date of dispensing, and a drug identifier (all provided National Drug Code and generic names). Some HMOs provided data for all CABG procedures. Others provided data only for patients whose CABG procedure was performed at an Epicenter hospital. However, in those cases they provided follow-up data from any hospital. We tested the completeness of the claims files in several ways (e.g., we assessed the number of days per patient on which ambulatory services were provided and the number of diagnoses listed on such days). We excluded health plans from our main analyses if their data appeared to be incomplete. The specific reasons for exclusion are described.

For each patient, we identified all claims with any codes suggestive of surgical site infection (Appendix 1). We refer to the codes in Appendix 1 as indicators of infection. These codes included inpatient diagnoses of infection, ambulatory diagnoses of infection, procedure codes suggestive of infection, and antibiotics dispensed in the ambulatory setting. This list is a more general set of codes previously identified as being important (17,18); we added closely related codes that had not appeared in our earlier datasets. We also assigned an estimated probability that infection had occurred by using a previously described algorithm developed in a dataset that included a broad range of surgical procedures, including coronary artery bypass (17,18, Appendix 2). Because the algorithm assigns a higher baseline probability of infection to patients undergoing cardiac surgery than those undergoing other procedures, 536 (97%) of 550 patients with any of the indicators in Appendix 1 had an estimated probability of infection exceeding 9.5%. Although we sought to confirm the infection status of the 536 with probabilities exceeding 9.5%, we refer to them as patients with any indicator, since this has a functionally equivalent meaning for patients undergoing CABG. Identifying patients with any indicator is much simpler than identifying patients who exceed a threshold predicted probability of infection.

#### Medical Record Information

We attempted to obtain the medical records of patients with claims with an indicator of infection. Because most patients received care from a variety of providers in different facilities, we requested the record of the first provider or facility that submitted a claim with an indicator of infection. In many cases, identifying an institution to request records from was not possible. In these cases, we requested the record the patient’s primary care physician. For logistical reasons, reviewing records of patients belonging to one of the health plans was not possible.

Patients’ records with an indicator code were reviewed by trained abstractors for evidence of postoperative surgical site infections, by using the criteria from the Centers for Disease Control and Prevention’s National Nosocomial Infection Surveillance System (CDC NNIS) (19). The reviewers also noted if the information in the medical record was adequate to judge the presence of infection. Typical reasons that records were judged inadequate included inappropriate date range of the records provided or lack of indication that the patient had received postoperative care from the provider. An infectious disease specialist reviewed records with evidence of abnormal wound healing and classified the outcome as confirmed infection, abnormal wound that met some criteria for infection, or no evidence of infection.

#### <H3>Chronic Disease Score

We computed a chronic disease score (the Clark TC score) (20,21) for each patient, for use as a comorbidity adjuster. Components of the chronic disease score include the patient’s age, sex, and prescription medications during the previous 6 months. Points are assigned for 29 diseases or disease categories (i.e., diabetes, if the patient has any dispensing of a drug typically used to treat the disease). The chronic disease score has been shown to predict hospitalization and also to correlate the risk for postoperative surgical site infection in a general surgery population (22,23).

### Data Analysis

Simple comparisons of categorical data were performed by chi-square testing. Continuous variables were often not normally distributed and were compared by nonparametric tests, either the Wilcoxon if two groups were being compared or the Kruskal-Wallis test for more than two. The strength of correlation between continuous variables was assessed with the Spearman correlation coefficient. Logistic regression was used to investigate a central question in the current investigation (i.e., if enough consistency existed between plans to allow comparisons between hospitals). This question was assessed by using a hospital-by-plan interaction term in the model. The model also assessed and controlled for the relative contribution of health plan, hospital, age, sex, and patient coexisting condition to the probability of individual patients having a claim suggestive of infection.

## Results

These health plans provided data for 3,014 CABG procedures performed from January 1996 through March 1999. A total of 858 patients had no claims for prescription drugs for 180 days before surgery, 46 had claims in two different claims systems, 39 had an uncertain procedure type, and 7 had a second CABG within 30 days. In addition, one of the plans was unable to provide claims for postoperative ambulatory care for 99% of its patients; claims from this plan were determined to be unusable because they were incomplete. All 252 persons represented by this claim system were excluded. We excluded the 1,061 patients with at least one of the criteria from our main analyses (some patients met more than one exclusion criterion). The total number of included procedures was 1,953, representing 65% of all procedures. The median age was 61 years, 78% were men, a median of 15 prescriptions were filled 6 months before surgery, and the median chronic disease score was 2,283 (Table 1). Postoperatively, a median of five prescriptions were filled in 30 days after surgery. Substantial differences existed between the health plans in members’ age, sex, chronic disease score, and number of prescriptions before surgery. In the 30 days after surgery, substantial differences existed in the number of prescriptions (all drugs) and number of days that patients received ambulatory care.

**Table 1:** Characteristics of the population of patients of five health-care plans in Massachusetts, January 1996–March 1999

	Plan 1	Plan 2	Plan 3	Plan 4	Plan 5	All	p value^a^
Procedures	161	584	635	363	210	1,953	
Age (range)^a^	67 (61–73)	59 (53–64)	60 (54–65)	59 (53–63)	72 (68–75)	61 (55–67)	<0.0001
Sex, % male	77	78	83	80	60	78	<0.0001
Prescriptions (range) during the 180 days before surgery^b^	14 (9-22)	18 (10-28)	18 (12-27)	9 (6-12)	14 (7-21)	15 (9-24)	0.0001
Chronic disease score (range)^b^	2,474 (1,774–3,704)	2,211 (1,403– 3,456)	2,378 (1,506–3,662)	2,156 (1,372–3,350)	2,369 (1,432–3,376)	2,283 (1,446–3,511)	0.01
Prescriptions (range) during the 30 days after surgery ^b^	5 (3–7)	6 (4–8)	4 (3–6)	5 (3–7)	3 (0–5)	5 (3–7)	<0.0001
Days (range) with ambulatory care claims ^b^	1 (1–2)	10 (7–13)	6 (3–8)	11 (8–16)	14 (10–18)	8 (5–13)	0.0001
Diagnoses on days with ambulatory claims (average)	2.6	2.6	2.2	1.9	1.5	2.2	
Patients with specified indicator of infection % (no. of patients):							
Diagnosis during index hospitalization	6 (9)	3 (17)	2 (14)	2 (7)	1 (2)	3 (49)	0.05
Diagnosis in ambulatory setting (excludes emergency room)	12 (19)	15 (89)	17 (105)	11 (39)	13 (28)	14 (280)	NS
Diagnosis in emergency rooms	2 (3)	3 (19)	2 (12)	2 (6)	1 (3)	2 (43)	NS
Diagnosis during second hospitalization, includes extended care facilities	9 (14)	7 (41)	2 (15)	2 (6)	0 (1)	4 (77)	<0.01
Antistaphylococcal antibiotic in ambulatory setting	16 (26)	18 (107)	13 (81)	14 (52)	12 (25)	15 (291)	0.05
Wound culture performed	2 (3)	2 (12)	5 (33)	6 (22)	1 (2)	4 (72)	<0.01
Wound care	0 (0)	1 (4)	2 (15)	1 (5)	1 (2)	1 (26)	0.052
^a^Kruskal-Wallis or chi-square tests of the null hypothesis of no difference between plans. ^b^Median (interquartile range).							

**Figure 1:**
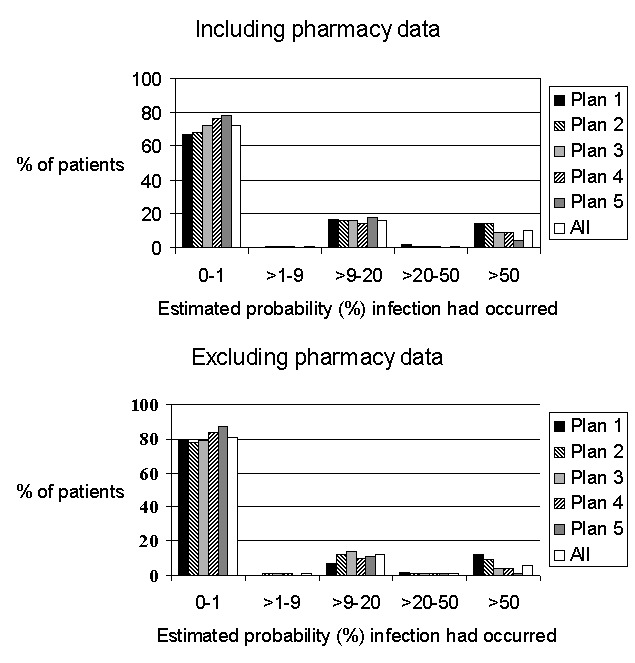
Distribution of patients’ probability of infection.

Overall, based on claims data alone, at least one indicator code for surgical site infection was found in 536 (27%) of 1,953 patients, with a range of 22% to 33% in the different health plans (Table 2). In patients with at least one such indicator code, the estimated probability that infection had occurred, based on our algorithm, was tightly clustered in two ranges: one was approximately 10% and the other approximately 70%. The distribution of estimated probabilities for all patients together and separately by health plan is shown in Figure 1. The overall pattern was similar across the health plans, although the distribution of probabilities was significantly different among them (p<0.01, Kruskal-Wallis). The specific types of indicators that contributed to patients being classified as high risk and the locations in which they occurred are shown in Table 1. Forty-nine (3%) patients had an infection indicator code during initial hospitalizations. In the 30 days after surgery, 77 (4%) persons had an indicator during a second hospitalization; 48 (62%) of second hospitalization occurred at the same institution in which surgery had been performed. Forty-three (2%) patients had an infection indicator during an emergency room visit, 280 (14%) had an indicator during an ambulatory-care visit, and 291 (15%) were dispensed an antistaphylococcal antibiotic (Table 1). Statistically significant differences occurred across health plans in the percentages of patients who had indicator diagnoses during initial hospitalization (p=0.05) and rehospitalization (p<0.01), who had claims for wound cultures (p<0.01) and wound care (p=0.052), and who received antistaphylococcal antibiotics (p=0.05) but not in diagnoses in emergency rooms or other ambulatory settings.

**Table 2:** Patients with indicators of infection by hospital and health maintenance organization claims system

Hospital	Plan 1	Plan 2	Plan 3	Plan 4	Plan 5	Total
	(% Patients with indicator/all patents)					
A	33 (36/108)	33 (45/136)	27 (48/175)	23 (12/53)	16 (7/45)	29 (148/517)
B	—	15 (7/41)	21 (54/256)	16 (21/131)	25 (8/32)	19 (89/460)
C	30 (3/10)	56 (9/16)	26 (16/62)	25 (12/48)	11 (3/28)	26 (53/164)
D	100 (1/1)	41 (41/100)	55 (50/141)	34 (29/86)	34 (11/32)	36 (132/360)
Other	31 (13/42)	28 (81/291)	0 (0/1)	27 (12/45)	25 (18/73)	27 (124/452)
All	33 (53/161)	31 (182/584)	26 (168/635)	23 (86/363)	22 (47/210)	27 (536/1953)

We requested full text medical records that had infection indicator codes for 368 patients who were members of plans that participated in the record review component of this study. We obtained 275 (75%) of these (Table 3). The health plan with automated ambulatory medical records retrieved nearly all requested records; from the others, the proportions ranged from 66% to 79%. From records obtained, 149 (54%) contained sufficient information to allow assessment of the presence or absence of postoperative surgical site infection. Common reasons for classifying the documentation inadequate were lack of any evidence that the provider or facility had cared for the patient during the 30 days after surgery or submission of records from a time that excluded this interval. Records were considered to be adequate if the status of the incisions during a postoperative visit was not mentioned. From charts with adequate documentation, 79 (53%) patients were confirmed to have infection; 70 of these infections were superficial. Another 19 (13%) patients partially satisfied CDC NNIS criteria for surgical site infection. The confirmation rate was similar for those with estimated probabilities of infection of >9%–20% (48%, 35/73) and those with estimated probabilities >50% (59%, 43/73). No substantial difference existed between either health plans or hospitals in the proportions with confirmed infection; these proportions exceeded 50% for every health plan and hospital except one, for which the confirmation rate was 45% (data not shown).

**Table 3:** Infections noted in full text medical record review^a^

	Plan 1	Plan 2	Plan 4	Plan 5	All
Records sought	53	182	86	47	368
Records obtained (%)	51 (96)	125 (69)	68 (79)	31 (66)	275 (75)
Adequate documentation (% of records received)	45 (88)	62 (50)	29 (43)	13 (42)	149 (54)
Adequate documentation among records sought	45 (85)	62 (34)	29 (34)	13 (28)	149 (40)
Surgical site status (% of those with adequate documentation)					
Confirmed surgical site infection	23 (51)	31 (50)	15 (52)	10 (77)	79 (53)
Problem wound healing, not meeting criteria for infection.	7 (16)	7 (11)	3 (10)	2 (15)	19 (13)
No evidence of infection	15 (33)	24 (31)	11 (38)	1 (8)	51 (34)

^a^For logistical reasons, records were not sought from plan 3.

**Figure 2:**
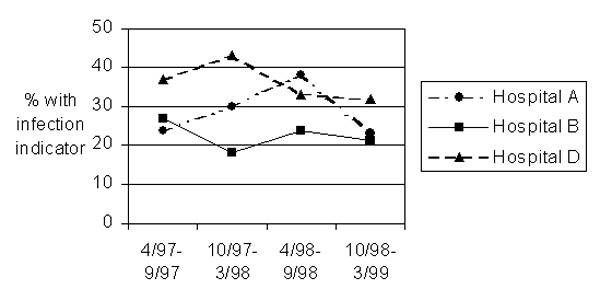
Proportion of patients with an indicator of infection, by hospital and 6-month period.

Of the four studied hospitals, the proportions of patients with an indicator of infection varied from 19% to 36% (Table 2). The rank ordering of the hospitals was consistent across the different health plans, with hospital D having the highest proportion in four plans and hospital B having the lowest in three of them. Hospital D’s excess, compared to hospitals A and B, was considerably greater during the year beginning April 1997 than during the year beginning April 1998 (Figure 2). Hospital B had either the lowest percentage or was close to the lowest during the 2 years. After these results were known, hospital D indicated that it had identified an increase in its sternal surgical site infection rate from July through December 1997 through hospital-based surveillance and had intervened to address this increase (pers. comm.,hospital D’s epidemiologist). The increase noted by hospital D overlapped with the two periods during which claims-based surveillance showed the hospital’s rate to be high.

The hospitals were different from one another in the proportion of patients with an indicator of infection (p<0.0001), after controlling for health plan, patient age, and patient sex. The adjusted relative odds of a patient’s having an indicator for infection for hospital D compared to hospital B was 2.3, with intermediate values for the others (Table 4). Patient age, sex, and health plan were also significant predictors of an indicator of infection. However, the interaction between health plan and hospital was not significant, indicating that the risk of infection at each hospital was not affected by membership in any particular plan. The adjusted relative risks for the hospitals were nearly identical in models that substituted the chronic disease score, which incorporates preoperative prescription drug information along with age and sex, in place of age and sex as separate risk factors. Nearly identical results were obtained when the health plans with highest and lowest values were excluded from these analyses, either singly or together. The results were also nearly the same when we included the 969 patients (totaling 2,922 of the original 3,014 cases) who had been excluded because they had no pharmacy dispensing activity during the 6 months before surgery or because they belonged to the health plan that provided no claims for postoperative ambulatory care.

**Table 4:** Adjusted hospital specific risks^a^

	Patients with at least one indicator code for infection	Hospital B vs. A	Hospital C vs. A	Hospital D vs. A	Other hospitals vs. A	p value
Including pharmacy data	536/1,953	0.68 (0.49–0.94)^b^	1.03 (0.68–1.55)	1.57 (1.16–2.13)	0.91 (0.67–1.24)	<0.0001
Excluding pharmacy data	363/1,953	0.84 (0.58–1.20)	0.92 (0.56–1.50)	1.62 (1.15–2.28)	1.05 (0.74–1.50)	0.03

^a^Adjusted for health plan, age, and sex. The interaction between health plan and hospital was not significant in any of these models. Similar results were obtained in models adjusting for chronic disease score (a composite of age, sex, and pharmacy information), instead of age and sex. ^b^Adjusted odds ratios and (95% confidence intervals).

We also assessed the effect of ignoring postoperative antibiotic information. In this situation, 363 (18%) of 1,953 persons had an indicator suggestive of infection, compared to 27% when antibiotics were included, using the same model and setting the contribution of absolute zero. The distribution of estimated probability that infection had occurred still had two peaks, clustered as before at 10% and 70% (Figure 1). These estimated probabilities including and excluding postoperative antibiotic information were highly correlated. For all patients together, the Spearman correlation coefficient was 0.81 (p=0.0001); the health plan specific correlation coefficients ranged from 0.71 to 0.83. When postoperative antibiotic dispensing in the ambulatory setting was ignored, qualitatively similar results regarding the relative odds associated with specific hospitals were also obtained, although the effect of the hospital was less strong (p=0.03, Table 4).

## Discussion

These results agree with earlier findings indicating the value of using automated claims data to identify persons who are likely to have experienced a postoperative surgical site infection. In this setting, infection was confirmed in approximately 58% of patients with an indicator in claims data. These findings were also consistent with earlier findings that most infections are detected in ambulatory settings or in hospitals other than the one in which surgery was performed. This result is notable in this case because complications of CABG are probably more likely to be cared for in the institution where surgery is performed, compared to complications of other types of procedures.

A principal reason to use automated data in this way would be to screen institutions periodically to identify those with higher than expected proportions of patients with an indicator of infection. However, a high proportion of patients with indicators does not necessarily imply that a hospital’s infection rate is high, since the overall confirmation rate may vary across hospitals and over time. Rather, the finding of a high proportion with infection indicator codes would allow directed inquiry about whether these institutions’ actual infection rates exceeded either their own usual level or the rates for similar institutions. In our data, the claims data suggested that hospital D’s rate was high, which was confirmed by the hospital. These data might also be used to identify institutions with consistently low proportions of patients with indicator codes; these institutions may be able to assist others in identifying and implementing best practices. In our case, hospital B may be such an institution, since its proportion of patients with an indicator code was usually the lowest of the group.

Since claims like the ones used here are created for nearly all patients, performing such screening for most hospitals would be possible. The type of work involved, manipulation of automated claims data and review of selected records, is similar to work already performed by many health maintenance organizations as part of their accreditation requirements. Such activities might be integrated with those of peer review organizations, which have experience in working with hospitals to assess care and to implement changes to improve it. The incremental work for health plans of performing this screening is relatively small, after the initial work of implementation.

The data we used were created mainly to support financial operations; therefore, the underlying data systems differ considerably within and between health plans. Health plans’ data systems differ (e.g., the number of diagnoses and procedures per claim that they capture). Additionally, the reliability of data can vary in ways not appreciated by the health plans themselves. For example, we found that one health plan identified no claims for postoperative ambulatory care. Limiting assessment to patients and procedures for which the overall patterns of care appear to be appropriate is important.

Health plans themselves differ in a variety of ways that may influence the proportion of patients with indicators for infection, including the patient populations they serve. These populations may differ in their underlying risk for infection. In the health plans we studied, the different distributions of age, sex, and chronic disease scores illustrate this point. The different benefits packages of the health plans also cause differences in the proportion with indicators. For example, one of the health plans had limited prescription drug coverage. Therefore, comparisons between health plans’ results must be made with care.

The differences between the health plans did not affect our inter-hospital comparisons, shown in Table 4. We interpret this result to mean that combining results from different health plans is possible, as long as the comparisons control for health plan. Combining results to obtain sufficient numbers of procedures and stable estimates of risk is desirable. Consistency of effect across insurers provides additional support for the comparisons.

Differences between hospitals’ proportions of patients with infection indicator codes may reflect a difference in the actual risk of infection, but they may also result from systematic differences in the way they or their clinicians assign diagnosis and procedure codes or report them to payers. Because of these potential differences, outlier values observed in claims data should be confirmed by direct assessment of clinical outcomes. For this reason and others, a hospital’s proportion of patients with indicator codes should not be equated with its infection rate.

Because our record review confirmed similar proportions of infections among patients with low and high estimated risks of infection, we recommend focusing on all patients with any of the indicators, rather than those with higher estimated probabilities of infection. Focusing on all patients with an indicator of infection eliminates some of the potential sources of uninformative variation between hospitals and health plans, since the estimation of risk for each patient uses combinations of specific codes. In addition, the larger number of events provides more stable estimates and therefore facilitates comparisons. The relatively small number of confirmed deep infections prevented assessment of differential ability of this method to identify different types of surgical site infections. We do not know whether the fact that 89% of confirmed infections were superficial reflects the actual epidemiology of surgical site infections or differential ascertainment of deep infections, which are likely to be diagnosed and treated in the inpatient setting. Our earlier work (18) suggested the approach we used had good sensitivity for detecting infections diagnosed among inpatients.

Although pharmacy claims are an indicator of infection, sometimes the only indicator, we obtained qualitatively similar results when we ignored this information. Therefore, we believe claims data can be assessed even when pharmacy claims are unavailable. However, controlling for the availability of this information will be important when making comparisons. Although automated ambulatory medical records are still not widely used, using their information when it is available is worthwhile, since this information is typically more complete. The same caveat will apply about controlling for automated medical records versus claims data as the data source.

We cannot directly extrapolate these results regarding coronary artery bypass to other types of procedures. For instance, ICD9 code 998.5 (postoperative infection) may be much less specific when assigned during hospitalizations for other types of surgical procedures. For example, the code may be assigned during a hospitalization for breast surgery to treat pre-existing infection. Because of this, the usefulness of these indicators for common procedure types should be determined. However, earlier work did indicate usefulness of indicators obtained from automated data for an unselected set of nonobstetric procedures (17) and separately for identification of postpartum infection (23).

We conclude that automated claims systems currently maintained by most health plans and insurers to reimburse institutions, providers, and pharmacies can serve as the basis for a screening system that would allow assessment of most hospitals’ outcomes of coronary artery bypass procedures and possibly of other procedure types. Such screening would allow focused follow-up of specific institutions to determine if their infection rates actually are high and if specific practices can be changed to reduce the risk to patients. Such screening systems will be particularly useful if different health plans combine their results. The effort required to implement a system that includes a majority of hospitals that perform coronary artery surgery is not large in relation to existing quality improvement programs and would provide information that complements existing programs for identification and control of surgical site infections.

## Supplementary Material

Appendix 1 Antibiotics, diagnosis, and procedure codes used to identify potential infections

Appendix 2 Algorithm used to assign probability that infection had occurred
