# Supplementary material for: Using Automated Health Plan Data to Assess Infection Risk from Coronary Artery Bypass Surgery
Source: Emerg Infect Dis. 2002 Dec;8(12):1433–41. doi: 10.3201/eid0812.020039 (PMC2737830; doi:10.3201/eid0812.020039)
Supplement: Appendix 1 — Antibiotics, diagnosis, and procedure codes used to identify potential infections [file 02-0039-app-s1.pdf]

Publisher: CDC; Journal: Emerging Infectious Diseases
Article Type: Research; Volume: 8; Issue: 12; Year: 2002; Article ID: 02-0039
DOI: 10.3201/eid0812.020039; TOC Head: Research

Appendix 1. Antibiotics, diagnosis, and procedure codes used to identify potential infections[a]

Antibiotics[b]

Cephalexin
Dicloxacillin
Clindamycin
Cephradine
Vancomycin

Diagnoses assigned in hospitals, emergency departments, or outpatient settings (ICD9 codes)

998.0	Postoperative Shock
998.3	Post-op Wound Disruption
998.5	Postoperative Infection
998.51	Infected Post-op Seroma
998.59	Post-op Infection Nec
998.83	Non-Healing Surg Wnd
780.6	Fever
891.0	Op Wnd Low Leg /S Comp
891.1	Open Wnd Knee/Leg-Comp
682.6	Cellulitis of Leg
682.9	Cellulitis Nos
998.9	Surgical Comp Nos
38.0	Streptococcal Septicemia
38.1	Staph Septicemia
38.10	Staph Septicemia Nos
38.11	Staph Aureus Septicemia
38.19	Staph Septicemia Nec
38.2	Pneumococcal Septicemia
38.3	Anaerobic Septicemia
38.4	Gram-Neg Septicemia Nec
38.40	Gram-Neg Septicemia Nos
38.41	*H. influenzae* Septicemia
38.42	*E. coli* Septicemia
38.43	Pseudomonas Septicemia
38.44	Serratia Septicemia
38.49	Oth Gram-Neg Septicemia
38.8	Septicemia Nec
38.9	Septicemia Nos
790.7	Bacteremia
611.0	Inflam Disease of Breast
682.0	Cellulitis of Face
682.1	Cellulitis of Neck
682.2	Cellulitis of Trunk

Publisher: CDC; Journal: Emerging Infectious Diseases
Article Type: Research; Volume: 8; Issue: 12; Year: 2002; Article ID: 02-0039
DOI: 10.3201/eid0812.020039; TOC Head: Research

682.3	Cellulitis of Arm
682.4	Cellulitis of Hand
682.5	Cellulitis of Buttock
682.6	Cellulitis of Leg
682.7	Cellulitis of Foot
682.8	Cellulitis, Site Nec
682.9	Cellulitis Nos
686.0	Pyoderma
686.1	Pyogenic Granuloma
686.8	Local Skin Infection Nec
686.9	Local Skin Infection Nos
958.3	Posttraum Wnd Infect Nec
711.00	Pyogen Arthritis-Unspec
996.6	Infect/Inflam-Dev/Graft
996.60	Infect Due To Device Nos
996.61	Infect D/T Hrt Device
996.62	Infect D/T Vasc Device
996.63	Infect D/T Nerv Device
996.64	Infect D/T Urethral Cath
996.65	Infect D/T GU Device Nec
996.66	Infect D/T Joint Prosth
996.67	Infect D/T Orth Dev Nec
996.68	Infect D/T PD Cath
996.69	Infect Due To Device Nec
674.3	Oth Comp OB Surg Wound
879.0	Open Wound of Breast
879.1	Open Wound Breast-Comp
879.2	Opn Wnd Anterior Abdomen
879.3	Opn Wnd Ant Abdomen-Comp
879.4	Opn Wnd Lateral Abdomen
879.5	Opn Wnd Lat Abdomen-Comp
879.6	Open Wound of Trunk Nec
879.7	Open Wnd Trunk Nec-Comp
879.8	Open Wound Site Nos
879.9	Opn Wound Site Nos-Comp
875.0	Open Wound-Chest/S Comp
875.1	Open Wound Chest-Comp

Specimens obtained for culture (CPT codes)

87040	Blood Culture for Bacteria
87072	Culture of Specimen by Kit
87075	Culture Specimen, Bacteria
87076	Bacteria Identification
87081	Bacteria Culture Screen
87082	Culture of Specimen by Kit

Publisher: CDC; Journal: Emerging Infectious Diseases
Article Type: Research; Volume: 8; Issue: 12; Year: 2002; Article ID: 02-0039
DOI: 10.3201/eid0812.020039; TOC Head: Research

87083	Culture of Specimen by Kit
87084	Culture of Specimen by Kit

Wound care procedures (CPT codes)

10180	Complex Drainage Wound
11000	Debride Infected Skin
11001	Debride Infect Skin Add
15852	Dressing Change, Not for Burn

[a]Some of these codes are applicable principally to surgical procedures other than CABG. They were included during the development of the algorithm shown in Appendix 2, which was developed to include CABG and other procedures [XXREF]. For consistency, these codes were retained in this evaluation of CABG procedures alone.
[b]These antibiotics were identified by an ambulatory pharmacy claim.
