# Supplementary material for: Using Automated Health Plan Data to Assess Infection Risk from Coronary Artery Bypass Surgery
Source: Emerg Infect Dis. 2002 Dec;8(12):1433–41. doi: 10.3201/eid0812.020039 (PMC2737830; doi:10.3201/eid0812.020039)
Supplement: Appendix 2 — Algorithm used to assign probability that infection had occurred [file 02-0039-app-s2.pdf]

Publisher: CDC; Journal: Emerging Infectious Diseases
Article Type: Research; Volume: 8; Issue: 12; Year: 2002; Article ID: 02-0039
DOI: 10.3201/eid0812.020039; TOC Head: Research

Appendix 2. Algorithm used to assign probability that infection had occurred

$p = \exp(\alpha + \beta_1 x_1 + \ldots + \beta_n x_{n)}/(1 + \exp(\alpha + \beta_1 x_1 + \ldots + \beta_9 x_9))$, where

$\alpha = –5.16$

$\beta_1 = +3.03$

x_1 = dispensing any of five selected antibiotics

$\beta_2 = +6.06$

x_2 = any selected diagnosis in hospital

$\beta_3 = +1.05$

x_3 = any selected diagnosis in emergency dept (if x2 = 0)

$\beta_4 = +2.98$

x_4 = any selected diagnosis in outpatient setting

$\beta_5 = +2.91$

x_5 = selected bacterial culture

$\beta_6 = +1.91$

x_6 = wound care

$\beta_7 = –1.79$

x_7 = interaction of x4 and x6

$\beta_8 = –2.70$

x_8 = interaction of x4 and x2

$\beta_9 = –2.21$

x_9 = interaction of x4 and x5
